# Molecular characterization and phylogenetic analysis of a *Squash leaf curl virus* isolate from Baja California Sur, Mexico

**DOI:** 10.7717/peerj.6774

**Published:** 2019-04-17

**Authors:** Diana Medina-Hernández, M. Goretty Caamal-Chan, Mayela Vargas-Salinas, Abraham Loera-Muro, Aarón Barraza, Ramón Jaime Holguín-Peña

**Affiliations:** 1 Programa de Agricultura en Zonas Áridas, Centro de Investigaciones Biológicas del Noroeste, La Paz, Baja California Sur, México; 2 Programa de Agricultura en Zonas Áridas, CONACYT-CIBNOR, Centro de Investigaciones Biológicas del Noroeste, La Paz, Baja California Sur, México

**Keywords:** Leaf curl disease, Iterons, Recombination, Genetic stability, Squash clade

## Abstract

**Background:**

The begomovirus, squash leaf curl virus (SLCuV) is one of the causal agents of squash leaf curl (SLC) disease, which is among the most destructive diseases of cucurbit crops in tropical, subtropical, and semiarid regions worldwide. This disease was originally reported in the American continent with subsequent spread to the Mediterranean basin. Up to now, SLCuV has only been detected by PCR in Mexico. This study provides the first complete sequence of a Mexican SLCuV isolate from Baja California Sur (BCS). In addition, the genome of the virus was characterized, establishing its phylogenetic relationship with other SLCuV isolates.

**Methods:**

The full genome (DNA-A and DNA-B) was amplified by rolling circle amplification, cloned and sequenced and the open reading frames (ORF) were annotated. Virus identification was performed according to the International Committee on Taxonomy of Viruses (ICTV) criteria for begomovirus species demarcation. To infer evolutionary relationship with other SLCuV isolates, phylogenetic and recombination analyses were performed.

**Results:**

The SLCuV-[MX-BCS-La Paz-16] genome (DNA-A and DNA-B) had 99% identity with SLCuV reference genomes. The phylogenetic analysis showed that SLCuV-[MX-BCS-La Paz-16] is closely related to SLCuV isolates from the Middle East (Egypt, Israel, Palestine and Lebanon). No evidence of interspecific recombination was determined and iterons were 100% identical in all isolates in the SLCuV clade.

**Conclusions:**

SLCuV-[MX-BCS-La Paz-16] showed low genetic variability in its genome, which could be due to a local adaptation process (isolate environment), suggesting that SLCuV isolates from the Middle East could have derived from the southwestern United States of America (USA) and northwestern Mexico.

## Introduction

Viruses of the genus *Begomovirus* (family *Geminividae*) are devastating pathogens that affect a variety of agronomic crops worldwide (Rojas et al., 2018). Begomoviruses are commonly associated with vegetables (Varma & Malathi, 2003) and have also been reported in medicinal and aromatic plants (Saeed & Samad, 2017). The genus *Begomovirus* has 388 species, which have importance by their worldwide distribution and their direct and negative impact over a wide range of crops (Zerbini et al., 2017). Begomoviruses can be divided based on their geographic location and genomic organization. In the Old World (OW) they can be mono- or bipartite and are often associated with DNA-satellites, while those in the New World (NW) are mostly bipartite (Rojas et al., 2005; Duffy & Holmes, 2007; Melgarejo et al., 2013). Two additional groups associated with a specific host instead of geographical location are the sweepoviruses (monopartite begomoviruses that affect sweet potato) (Trenado et al., 2011) and the legumoviruses (bipartite begomoviruses that affect legumes), constituting two divergent monophyletic groups distinct from OW and NW begomoviruses (Ilyas et al., 2009).

Squash leaf curl virus (SLCuV) is a typical NW, bipartite begomovirus which infects squash (*Cucurbita pepo* L.) in North America (Flock & Mayhew, 1981) and the Mediterranean basin (Antignus et al., 2003; Lapidot et al., 2014). SLCuV popoulation from the Middle East show a low degree of genetic variability (Lapidot et al., 2014), and there is little genetic differentiation between population from North America and the Middle East (Rosario et al., 2015). Although it has been found in mixed infections with other begomoviruses (Kuo et al., 2007; Sufrin-Ringwald & Lapidot, 2011; Ali, Mohammad & Khattab, 2012; Ahmad, Odeh & Anfoka, 2013), recombinants have not been detected (Rosario et al., 2015). However, the recent migration and rapid spread of the SLCuV from the Americas into the Middle East could influence the appearance of new virulent strains and the expansion of the host range of the virus in native flora (Abudy et al., 2010). Thus, surveillance is necessary to monitor the appearance of new strains. The objective of this study was to characterize a SLCuV isolate from Mexico to infer its phylogenetic and evolutionary relationships with other isolates.

## Materials and Methods

### Samples collection and DNA extraction

Plant samples of *Cucurbita pepo* L. showing the characteristics of SLC disease were collected. Samplings were performed in the most important squash crops in the southern part of Baja California Sur State (BCS) during the spring/summer and autumn/winter cycles from 2016 to 2017. Total nucleic acids were isolated using a CTAB method (cetyl trimethylammonium bromide) (Doyle, 1991).

### SLCuV PCR detection

To detect and identify SLCuV, samples were tested by PCR. One microliter of total DNA from each sample (50 ng/µL) was used as template. The reaction mixture consisted of 0.5 µM forward and reverse primers (SqA2F and SqA1R; Table 1), 10 µL of 2×Phusion High-Fidelity PCR Master Mix (New England Biolabs, Inc., Ipswich, MA, USA) in 20 µL of final reaction volume. The PCR reaction was carried out as follows: initial denaturation step (98 °C 30 s, one cycle), amplification step for 35 cycles (98 °C 10 s, 55 °C 30 s and 72 °C 30 s, for each cycle), and a final elongation step (72 °C, 5 min).

**Table 1:** Primers used for detection and assembly of the full-length (DNA-A and DNA-B) of the squash leaf curl virus (SLCuV) in Baja California Sur, Mexico.

Name primer	Sequence (5′–3′)	pb	Temp.	Reference
	Target DNA-A		
SLCVF-Sall	TATAGTCGACGTTGAACCGGATTTGAATG	2,667	57	Farrag et al. (2014)
SLCVR-Sall	TATAGTCGACCTGAGGAGAGCACTAAATC		
SqA2F	TATCTCCCATCTTGGCAAGG	601	55	Sobh et al. (2012)
SqA1R	AGCTGTATCTTGGGCAACAGA		
SLCVA2295F	CAGATAATTGAATGAGGCAG	1,500	57	Lapidot et al. (2014)
Xho-SLCV-R	TGTACTCGAGAATCATGAAATAAAATTC		
SLCVA2314R	CTGCCTCATTCATTCAATTATCTG	1,300	57	Lapidot et al. (2014)
Xho-SLCVA-F	CATGATTCTCGAGTACATAATTTAC			
	Target DNA-B			
SLCVDNAB1F	GTGGTTATGCAAGGCGTCGACCCAAC	1,316	57	Lapidot et al. (2014)
SLCVDNAB1R	GCAAACTGAAGCTATCGTCGGCGAAGC		
SLCVDNAB2F	GCTTCGCCGACGATAGCTTCAGTTTGC	1,644	57	Lapidot et al. (2014)
SLCVDNAB2R	GTTGGGTCGACGCCTTGCATAACCAC		
BgMP-BC1F	WGCAAGACTVARTCGWAGCTGYATGAA	600	55	This article
BgMP-BC1R	TTKRGCCCCHAYTATDGAAGCHGAM			

### SLCuV full-length genome amplification

From SLCuV positive samples, total DNA was used as template for rolling circle amplification (RCA) using the TempliPhi Kit (GE Healthcare, Chicago, IL, USA) following the manufacturer’s protocol. RCA amplification products were digested with restriction enzymes *Cla* I and *Xba* I (New England Biolabs) to linearize DNA-A and DNA-B, respectively. Both DNA-A and DNA-B linearized genomic segments were isolated and ligated into pGEM-T Easy vector (Promega, Madison, WI, USA) according to the manufacturer’s protocol, and then used to transform *Escherichia coli* DH5-α. Recombinant clones were Sanger sequenced bidirectionally using SLCVF-SalI, SLCVR-SalI, SLCVA2295F, XhoSLCVR, XhoSLCVAF, SLCVA2314R primers for DNA-A and SLCVDNAB1F, SLCVDNAB1R, SLCVDNAB2R, SLCVDNAB2F, BgMP-BC1F, BgMP-BC1R primers for DNA-B (Table 1).

### Genome assembly and annotation

The resulting Sanger sequencing reads were used to assemble the SLCuV-[MXBCS:La Paz-16] full genome. All reads of DNA-A and DNA-B were mapped with reference to a SLCuV isolate from Palestine (PAL) (KC441465 and KC441466, respectively). To assemble the genome and identify the open reading frames (ORFs) “Geneious mapper and ORF finder” of Geneious R10 bioinformatics suite (https://www.geneious.com) were used. In addition, the identified ORFs for both DNA-A and DNA-B segments were confirmed with Frame Plot v4.0 beta and Global Align programs (Ishikawa & Hotta (1999); https://www.ncbi.nlm.nih.gov/orffinder/). The DNA-A and DNA-B full sequences were aligned with 11 SLCuV isolates (Table S1) using the MUSCLE algorithm implemented in MEGA7 (Kumar, Stecher & Tamura, 2016). Virus identification was performed based on the DNA-A sequence, by pairwise comparison using Species Demarcation Tool (SDT) v2.0 (Muhire, Varsani & Martin, 2014), following the ICTV species demarcation criteria (Brown et al., 2015).

### Phylogenetic, recombination and iterons analysis

Phylogenetic analysis was performed using complete DNA-A and DNA-B nucleotide sequences as well as replication-associated (Rep) and capsid (CP) protein amino acid sequences (see Table S1 for details of the sequences used). Phylogenetic trees were constructed with MEGA7 (Kumar, Stecher & Tamura, 2016) using the Neighbor-Joining (NJ) algorithm with the Kimura 2-parameter substitution model and 1,500 bootstrap replications. The RDP4 program was used to identify putative recombination events (Martin et al., 2015). The comparative analysis of the conserved elements in the IR (Argüello-Astorga et al., 1994) was performed using Clustal X2 and MEGA7.

## Results

### Virus detection

**Figure 1:**
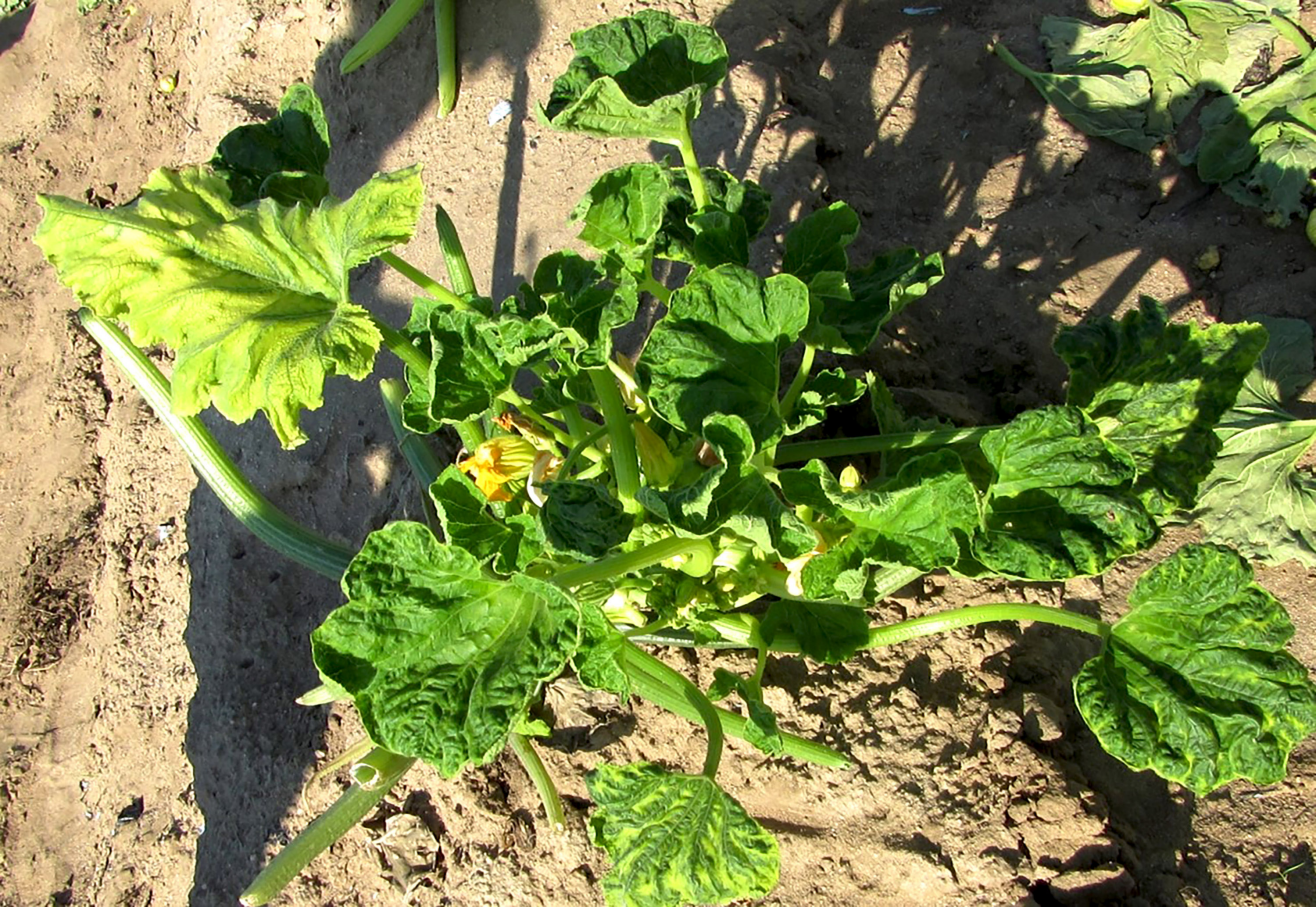
Symptoms associated to squash leaf curl virus (SLCuV) in *Cucurbita pepo*: thickened leaf vein-banding, mild chlorosis, severe leaf curling, reduction in the size of leaf, leaf distortion and mottled interveinal tissue were observed in squash plantation. Photograph by Mayela Vargas-Salinas.

A typification of the SLC disease was performed in the field observing symptoms of thickened leaf vein-banding, mild chlorosis, severe leaf curling, reduction in the size of leaf, leaf distortion and mottled interveinal tissue (Fig. 1). In preparation for viral detection, we performed a PCR-based detection with specific primers (SqA2F and SqA2R) obtaining the expected ∼600 pb size product.

### SLCuV genome annotation and identification

**Figure 2:**
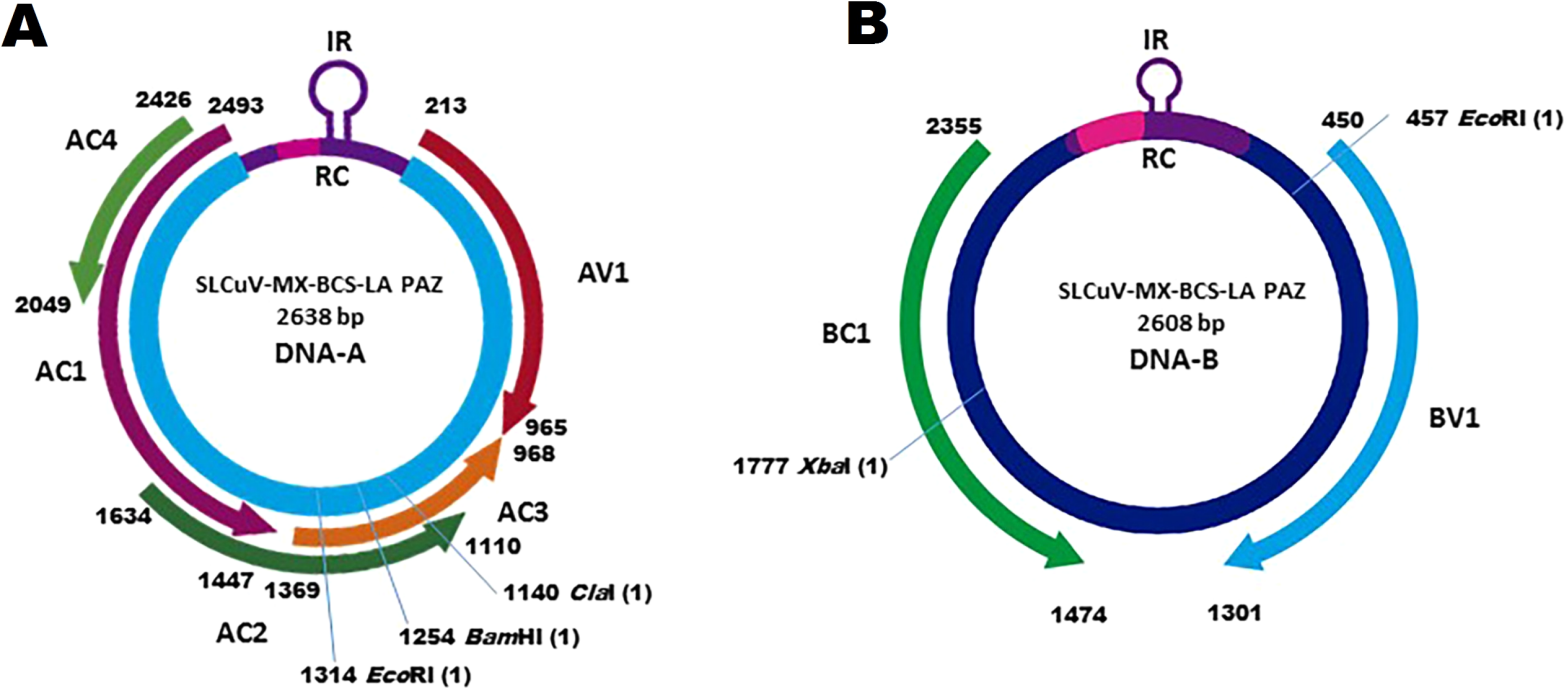
Genomic organization of the squash leaf curl virus Mexico (SLCuV-MX-BCS-La Paz-16) showing the predicted open reading frames (ORFs), size and position in the genomes. (A) DNA-A, (B) DNA-B. Arrows indicate direction in the viral sense (clockwise) and strained (anti-clockwise) direction. IR (intergenic region), CR (common region), ORFs in the DNA-A; AV1 (CP), AC1 (Rep), AC2 (Trap), AC3 (REn) and ORFs in the DNA-B; BV1 and BC1 (movement proteins) are represented in scale according to nucleotide size. The unique cutting by enzymes sites are shown in both genomes.

Several positive samples were analyzed by RCA to obtain both DNA components. DNA-A is 2,638 nt in length and contains five ORFs: AV1 (755 nt), AC1 (1,046 nt), AC2 (395 nt), AC3 (404 nt) and AC4 (377 nt) while DNA-B is 2,608 nt with two ORFs, BV1 (851 nt) and BC1 (881 nt) (Fig. 2). DNA-A ORFs have identity percentages ranging from 97 to 99% with other SLCuV isolates, while DNA-B ORFs have identities from 93 to 94%. In addition, lengths of the different ORFs are homogeneous ( ± 4 nt) when compared with other SLCuV isolates(Table 2). The highest identities for DNA-A, of 99% were with isolates from Egypt (DQ285019), Israel (HQ184436), Jordan (JX444577), Lebanon (HM368373) and Palestine (KC441465) (Table 2). Identities with isolates from southwest USA (M38183, DQ285016, AF256203) were of 98% (Table 2). Based on pairwise DNA-A sequence comparisons and following the species demarcation criteria for begomoviruses (Brown et al., 2015; Zerbini et al., 2017), the BCS isolate is a member of the species *Squash leaf curl virus* (SLCuV), with the acronym SLCuV-[MX-BCS-La Paz-16]. The sequences of SLCuV-[MX-BCS-La Paz-16] were deposited in GenBank with accession numbers MF187211 and MG544926 for the DNA-A and to DNA-B, respectively.

**Table 2:** Percentage nucleotide identity of squash leaf curl virus Mexico (SLCuV-[MX-BCS-La Paz-16]) isolated from infected squash in La Paz in the Peninsula of Baja California compared with the selected SLCuV clade members. ^1^ORF, Open Ready Frame. ^2^NA, Data not available.

Isolate	Access number	DNA-A	DNA-B	AV1	AC1	AC2	AC3	AC4	BV1	BC1
SLCuV-[US:IV:79]	M38183	98	94	92	NA1	98.8	98.1	NA	96.9	98.8
SLCuV-[US:IV:04]	DQ285016	98	94	98	99	99	98	98	93	97
SLCuV-[US:AZ:W:04]	AF256203	98	94	98	99	99	98	98	93	97
SLCuV-[EG:Cai:06]	DQ285019	99	NA	99	99	100	98	98	NA	NA
SLCuV-[JD:Mal:06]	EF532620	97	94	99	95	99	98	89	92	98
SLCuV-[IL:03]	HQ184436	99	NA	99	99	98	99	98	NA	NA
SLCuV-[EG:Ism:12]	KC895398	97	NA	99.6	95.1	98.7	97.3	91.8	NA	NA
SLCuV-[LB:09]	HM368373	99	94	99	99	100	98	99	99	99
SLCuV-[JO:Hor:11]	JX444577	99	93	99	99	99	98	99	99.2	98.6
SLCuV-[PL:10]	KC441465	99	NA	99	99	100	98	99	NA	NA
SLCuV-[JO:Sarv:11]	JX131281	99	93	99	98.6	100	98.3	98.4	96.9	98.9
MCLCuV-[CR:Gua:98]	AY064391	89	73	88	86	89	90	NA	83	67

### Phylogenetic analysis

**Figure 3:**
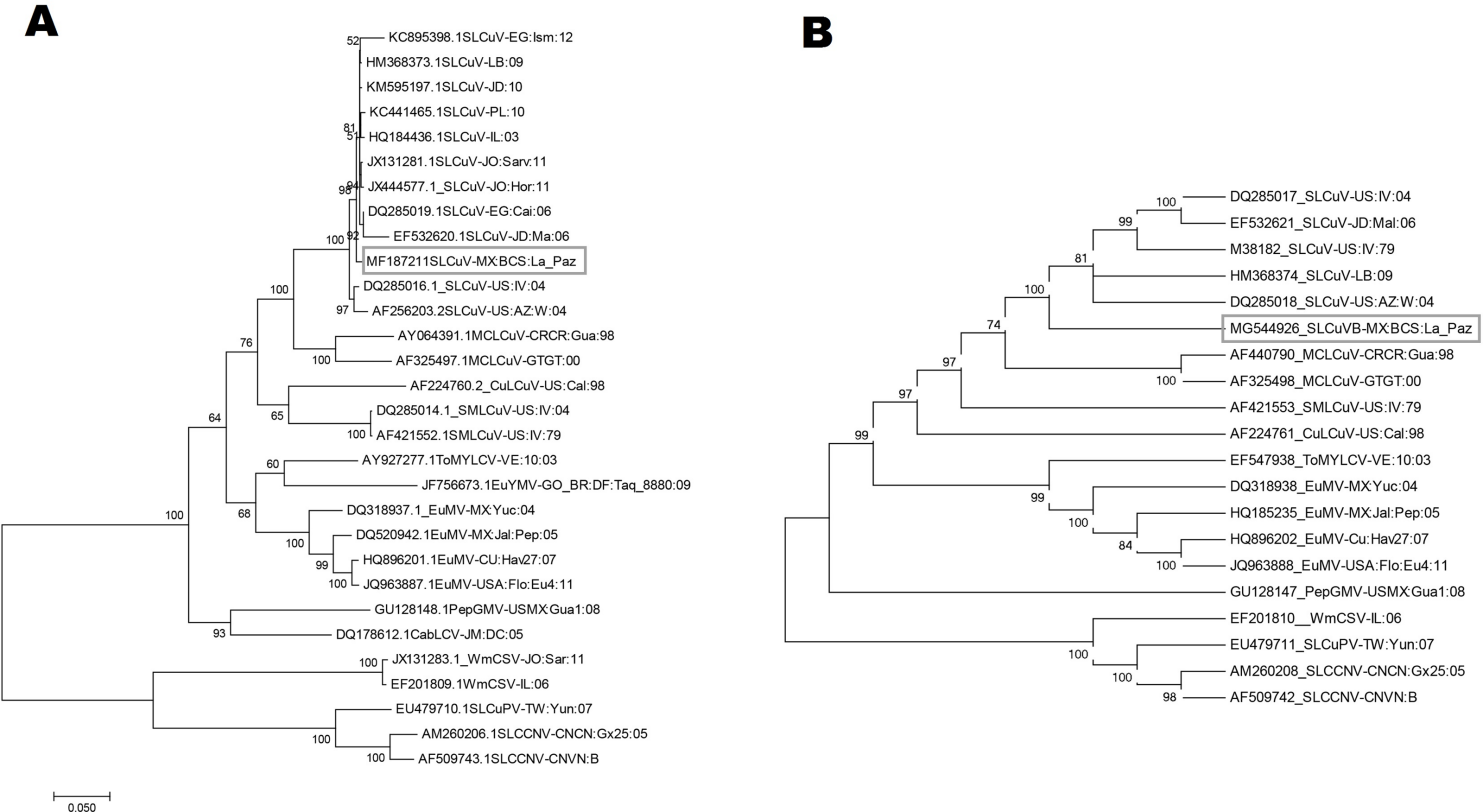
Phylogenetic tree showing the genetic relationship of the squash leaf curl virus Mexico with other begomoviral isolates from SLCuV, based full (A) DNA-A and (B) DNA-B. The values below node represent percentages of statistical support of evolutionary branch length in 1500 bootstrap replicates, branches with less than 60% bootstrap support have been collapsed.

The phylogenetic tree based on full-length DNA-A nucleotide sequences revealed that SLCuV-[MX-BCS-La Paz-16] forms a monophyletic group with other SLCuV isolates and a separate group with related NW begomoviruses that infect cucurbits and other hosts. SLCuV-[MX-BCS-La Paz-16] shows the closest relationship with SLCuV isolates from the Middle East, including Egypt, Lebanon, Palestine and Jordan (Fig. 3A). The phylogenetic analysis was well supported with high bootstrap values, and is consistent with pairwise sequence identity analyses. We carried out the same phylogenetic analysis with the DNA-B component (Fig. 3B), confirming the close phylogenetic relationship among SLCuV isolates. Phylogenetic trees based on amino-acid sequences of REP and CP also indicated that the SLCuV-[MX-BCS-La Paz-16] formed a single cluster with other SLCuV isolates (Fig. S1).

### Recombination and iterons analysis

In the analyses to search for potential recombination events in the DNA-A, we used the same data set used for the DNA-A phylogenetic analysis, including the NW and OW groups as well as other cucurbit begomoviruses. No putative recombination events were identified between SLCuV and other cucurbit begomoviruses. Using a second data set comprising only SLCuV isolates, two putative recombination events were supported by five of the seven different methods of the RDP package, indicating major parents Middle East isolates and the USA isolate US-AZ-04 as the minor parent. In the analysis of the intergenic region, the TAATATTAC sequence at the hairpin structure of geminiviruses was conserved in SLCuV-[MX-BCS-La Paz-16]. The analysis of the iterons located in the promoter region associated with the Rep protein showed four direct repeats and two inverted repeats, with 100% identity in the sequences of iterons with other SLCuV isolates (Fig. S2).

## Discussion

This study sequenced the full genome (DNA-A and DNA-B) of a SLCuV isolte from Mexico (SLCuV-[MX-BCS-La Paz-16]). It is worth noting that this is the first SLCuV full genome sequenced in Mexico, with all previous SLCuV detections having been limited to PCR-based diagnosis (Ramirez-Arredondo et al., 1995; Lugo et al., 2011). Despite the presence of SLCuV in North America and the Middle East, the genome seems to be very stable (Lapidot et al., 2014; Rosario et al., 2015) with no substantive changes in the sequence since the first genomic characterization of the virus (Cohen et al., 1983; Antignus et al., 2003). Our isolate is a typical SLCuV isolate with only slight modifications in the nucleotide sequence but without changes in the ORFs sizes and organization. The absence of genetic variations and the iteron analysis (without changes in sequence, number and orientation) is further evidence of the genomic stability observed in SLCuV-[MX-BCS-La Paz-16] with respect to other SLCuV isolates. SLCuV-[MX-BCS-La Paz-16] formed a discrete monophylogenetic group with the SLCuV clade but closer with the isolates from Middle Eastern countries (Egypt, Lebanon, Jordan and Palestine) than with the isolates from the USA. Despite the selection pressures, the interaction of the virus with the host and its vector and the biological-ecological interactions that confronts the viral populations, the genomic stability of the SLCuV seems to be maintained over time, preserving its genetic and structural functionality (Gibbs et al., 1999; Sánchez-Campos et al., 2002).

## Conclusion

The complete genome of SLCuV was sequenced for the first time in the Mexico, in the southern part of the Baja California peninsula. The molecular characterization indicated a closer relationship with isolates from Middle East rather than with isolates from the USA, suggesting that SLCuV might have reached BCS from the Middle East or vice-versa and not from the USA as it had been previously assumed. In order to confirm this hypothesis, phylogeographic studies should be performed to determine the paths of dispersion.

##  Supplemental Information

10.7717/peerj.6774/supp-1 Figure S1 Maximum likelihood phylogenetic (A) phylogenetic tree of replication associated capsid protein (CP) amino acid sequences, (B) protein (Rep) amino acid sequences The values below node represent percentages of statistical support of evolutionary branch length in 1500 bootstrap replicates, branches with less than 60 % bootstrap support have been collapsed.

10.7717/peerj.6774/supp-2 Figure S2 Phylogenetic tree showing the genetic relationship of the Squash leaf curl virus Mexico with other variants from SLCuV based full DNA-A and DNA-B The values below node represent percentages of statistical support of evolutionary branch length in 1,500 bootstrap replicates; branches with less than 60% bootstrap support have been collapsed. Figure 5 Analysis of iterons of Squash leaf curl virus Mexico (SLCuV-MX:BCS:La Paz). Showing the arrangement of the iterons 5′-GGTGTCC-3′in the viral sense and 5′-GGACACCA-3′in the complementary sense within of region CR; numbers 1–6 represent iterons; the number flanking the iterons indicates nucleotide location in the viral sense starting from the SCE in the stem-loop structure; the direction of four iterons upstream of a TATA of the AC1 initiation codon (see arrows) and two in inverted sense; found TATAA box of Rep in position 2569-2574 and GC box in position 10–15. All iteron elements were identical in all the SLCuV variants analyzed, including the SLCuV-MX:BCS:La Paz.

10.7717/peerj.6774/supp-3 Table S1 Information of selected squash leaf curl virus (SLCuV) sequences used in this study correspond to those sequences reported as species by the International Committee on Taxonomy of Viruses (ICTV) 1 Referenced as SLCuV species by the International Committee on Taxonomy of Viruses (ICTV; https://talk.ictvonline.org.) Abbreviation hots. Sq, Squash (*Cucurbita pepo*), BHR broad host range of SLCuV that are delimited mainly in five families; *Cucurbitaceae*, *Fabaceae*, *Malvaceae*, *Brassicaceae* and *Solanaceae* (Singh et al., 2008; Abudy et al., 2010; AliShtayeh et al., 2014; A Farrag et al., 2014), To, Tomato (Solanum lycopersicum), Chm, Charlock mustard (Sinapis arvensis), Wat, Watermelon (Citrullus lanatus), Cuc, Cucumber (Cucumis sativus), Cm, Honeydew melon (Cucumis melo), Mp, Malva parviflora.

## Additional Information and Declarations

### Data Availability

The following information was supplied regarding data availability: GenBank: MF187211, MG544926.
