# Supplementary figures and images for: Molecular characterization and phylogenetic analysis of a Squash leaf curl virus isolate from Baja California Sur, Mexico
Source: PeerJ. 2019 Apr 17;7:e6774. doi: 10.7717/peerj.6774 (PMC6475161; doi:10.7717/peerj.6774)

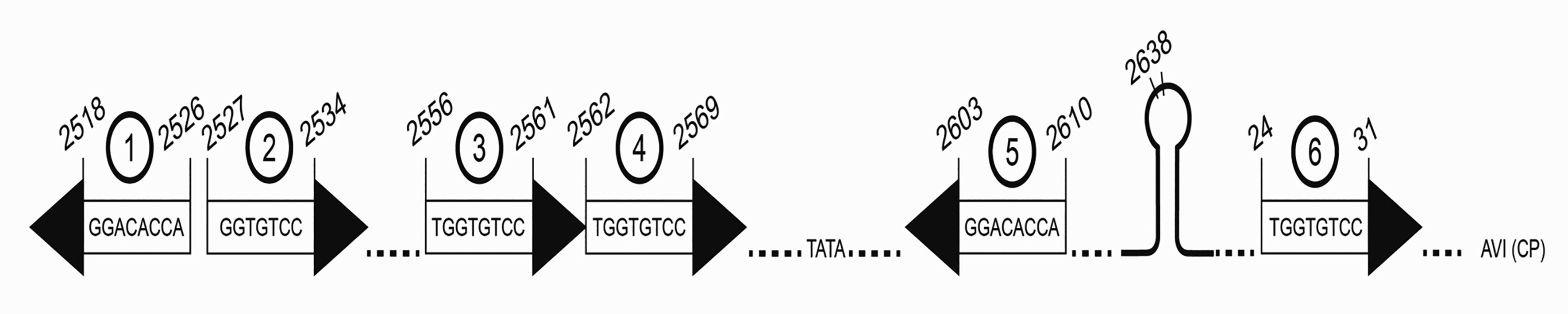

Supplement: Figure S2 — The values below node represent percentages of statistical support of evolutionary branch length in 1,500 bootstrap replicates; branches with less than 60% bootstrap support have been collapsed. Figure 5 Analysis of iterons of Squash leaf curl virus Mexico (SLCuV-MX:BCS:La Paz). Showing the arrangement of the iterons 5′-GGTGTCC-3′in the viral sense and 5′-GGACACCA-3′in the complementary sense within of region CR; numbers 1–6 represent iterons; the number flanking the iterons indicates nucleotide location in the viral sense starting from the SCE in the stem-loop structure; the direction of four iterons upstream of a TATA of the AC1 initiation codon (see arrows) and two in inverted sense; found TATAA box of Rep in position 2569-2574 and GC box in position 10–15. All iteron elements were identical in all the SLCuV variants analyzed, including the SLCuV-MX:BCS:La Paz. [file peerj-07-6774-s002.png]
