# Supplementary material for: Molecular characterization and phylogenetic analysis of a Squash leaf curl virus isolate from Baja California Sur, Mexico
Source: PeerJ. 2019 Apr 17;7:e6774. doi: 10.7717/peerj.6774 (PMC6475161; doi:10.7717/peerj.6774)
Supplement: Table S1 — 1 Referenced as SLCuV species by the International Committee on Taxonomy of Viruses (ICTV; https://talk.ictvonline.org.) Abbreviation hots. Sq, Squash (Cucurbita pepo), BHR broad host range of SLCuV that are delimited mainly in five families; Cucurbitaceae, Fabaceae, Malvaceae, Brassicaceae and Solanaceae (Singh et al., 2008; Abudy et al., 2010; AliShtayeh et al., 2014; A Farrag et al., 2014), To, Tomato (Solanum lycopersicum), Chm, Charlock mustard (Sinapis arvensis), Wat, Watermelon (Citrullus lanatus), Cuc, Cucumber (Cucumis sativus), Cm, Honeydew melon (Cucumis melo), Mp, Malva parviflora. [file peerj-07-6774-s003.docx]

Table S1. Information of selected squash leaf curl virus (SLCuV) sequences used in this study correspond to those sequences reported as species by the International Committee on Taxonomy of Viruses (ICTV).

| *Squash leaf curl virus* variants | Acronym | Host | Origin | GenBank accession no.  DNA-A | GenBank accession no.  DNA-B | Reference |
| --- | --- | --- | --- | --- | --- | --- |
| **In this study** |  |  |  |  |  |  |
| Squash leaf curl virus isolate Mexico:BCS | SLCuV-MX:BCS: La Paz | Sq | Mexico  (Baja California Sur) | MF187211 | MG544926 |  |
| **From GenBankdB^1^** |  |  |  |  |  |  |
| Squash leaf curl virus [United States:Imperial Valley:1979] | SLCuV-[US:IV:79] | BRH | USA (California) | M38183 | M38182 | Lazarowitz and, 1991 |
| Squash leaf curl virus - [United States:Imperial Valley:2004] | SLCuV-[US:IV:04] | Sq. | USA (California) | DQ285016 | DQ285017 | Brown et al., 2005b |
| Squash leaf curl virus - [United States:Arizona:W:2004] | SLCuV-[US:AZ:W:04] | Sq, Cm | USA (Arizona) | AF256203.2 | DQ285018 | Brown et al., 2000; 2005. |
| Squash leaf curl virus - [Egypt:Cairo:2006] | SLCuV-[EG:Cai:06] | Sq. | Egypt  (Cairo) | DQ285019 | DQ285020 | Idris et al., 2006 |
| Squash leaf curl virus - [Jordan:Malva:2006] | SLCuV-[JD:Mal:06] | Sq.  Mp. | Jordan | EF532620 | EF532621 | Almusa et al., 2008 |
| Squash leaf curl virus - [Israel:2003] | SLCuV-[IL:03] | Sq. | Israel | HQ184436 | NA | Abudy et al., 2010 |
| Squash leaf curl virus - [Egypt:Ismaili:2012] | SLCuV-[EG:Ism:12] | Sq. | Egypt  (Ismaili) | KC895398 | NA | El Reham., 2015 |
| Squash leaf curl virus - [Lebanon:2009] | SLCuV-[LB:09] | Sq. | Lebanon | HM368373 | HM368374 | Sobh et al., 2012 |
| Squash leaf curl virus - [Jordan:Homra:2011] | SLCuV-[JO:Hor:11] | To. | Jordan | JX444577 | JX444574 | Ahmad et al., 2013 |
| Squash leaf curl virus - [Palestine:2010] | SLCuV-[PL:10] | Sq. Wat., Cuc. | Palestine | KC441465 | NA | Ali-Shtayeh et al., 2014 |
| Squash leaf curl virus - [Jordan:Sinapis arvensis:2011] | SLCuV-[JO:Sarv:11] | Chm. | Jordan | JX131281 | JX131282 | Anfoka et al., 2017 |

^1^ Referenced as SLCuV species by the International Committee on Taxonomy of Viruses (ICTV; <https://talk.ictvonline.org>)

Abbreviation hots. Sq = Squash (*Cucurbita* *pepo*), BHR broad host range of SLCuV that are delimited mainly in five families; *Cucurbitaceae*, *Fabaceae*, *Malvaceae*, *Brassicaceae* and *Solanaceae* (Singh et al., 2008; Abudy et al., 2010; Ali-Shtayeh et al., 2014; A Farrag et al., 2014), To = Tomato (Solanum lycopersicum), Chm = Charlock mustard (Sinapis arvensis), Wat = Watermelon (Citrullus lanatus), Cuc = Cucumber (Cucumis sativus), Cm = Honeydew melon (Cucumis melo), Mp=Malva parviflora.
